# Supplementary figures and images for: Fatty Acid Synthesis Knockdown Promotes Biofilm Wrinkling and Inhibits Sporulation in Bacillus subtilis
Source: mBio. 2022 Sep 7;13(5):e01388-22. doi: 10.1128/mbio.01388-22 (PMC9600695; doi:10.1128/mbio.01388-22)

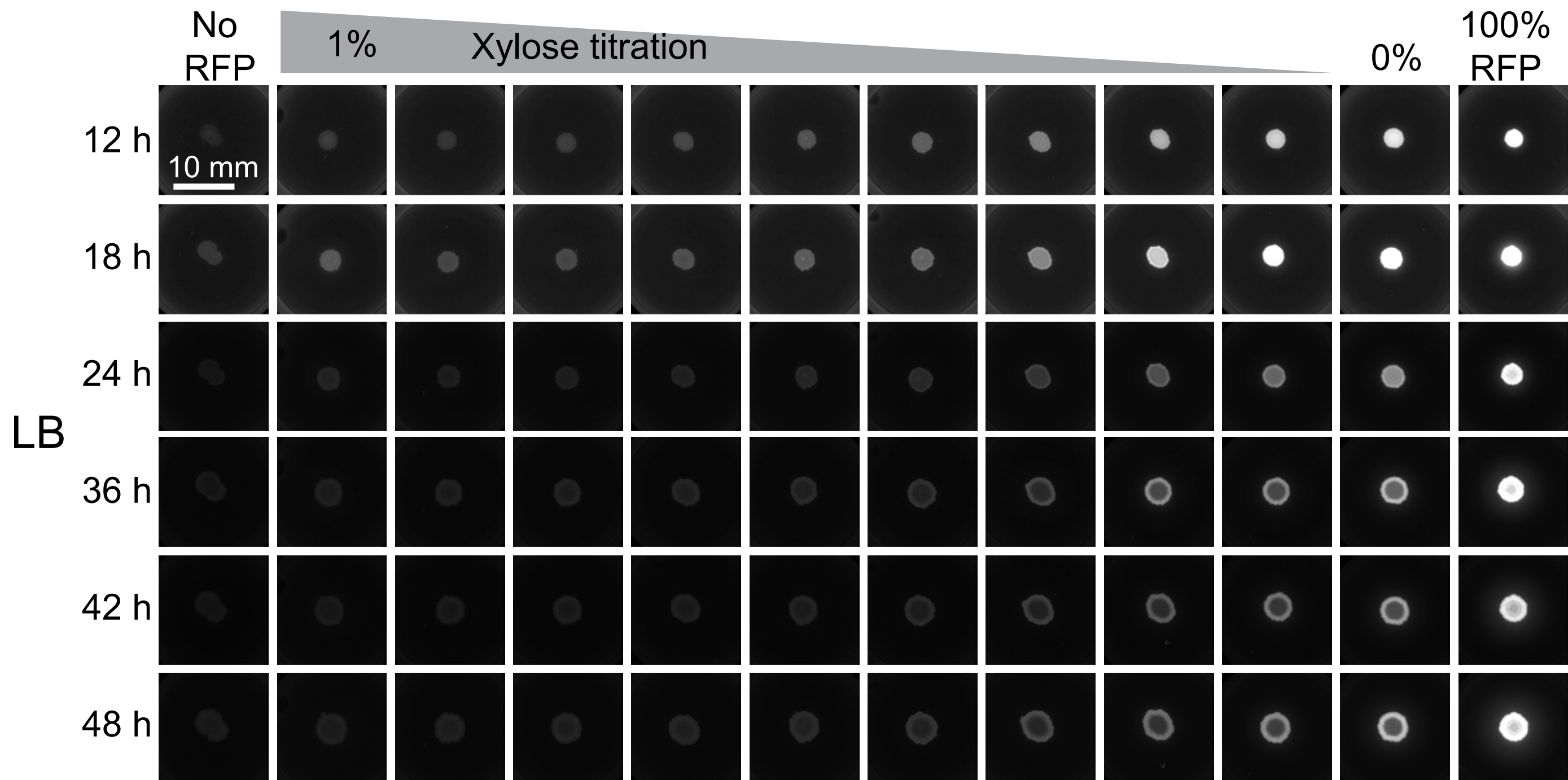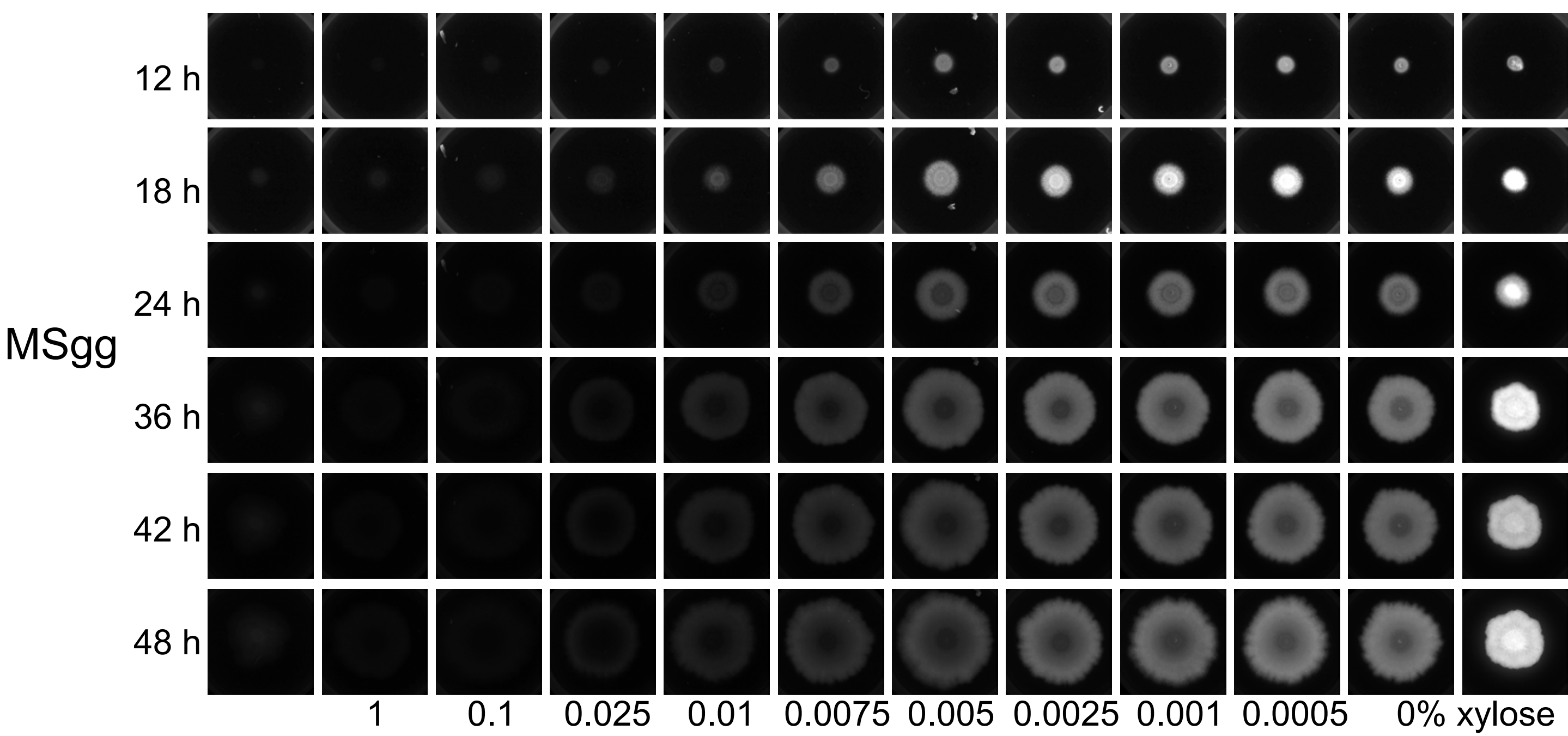

Supplement: FIG S1 [file mbio.01388-22-s0007.pdf]

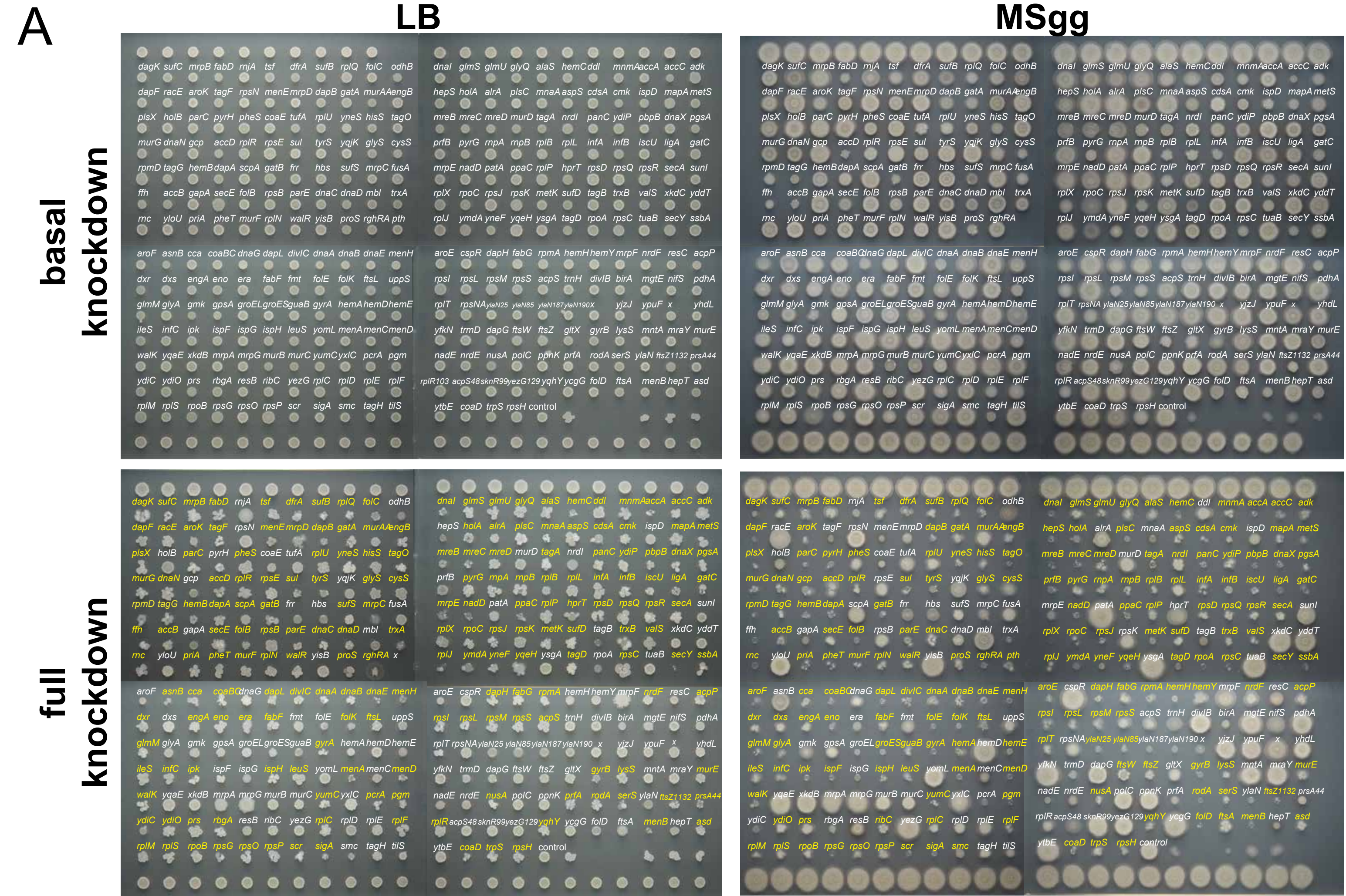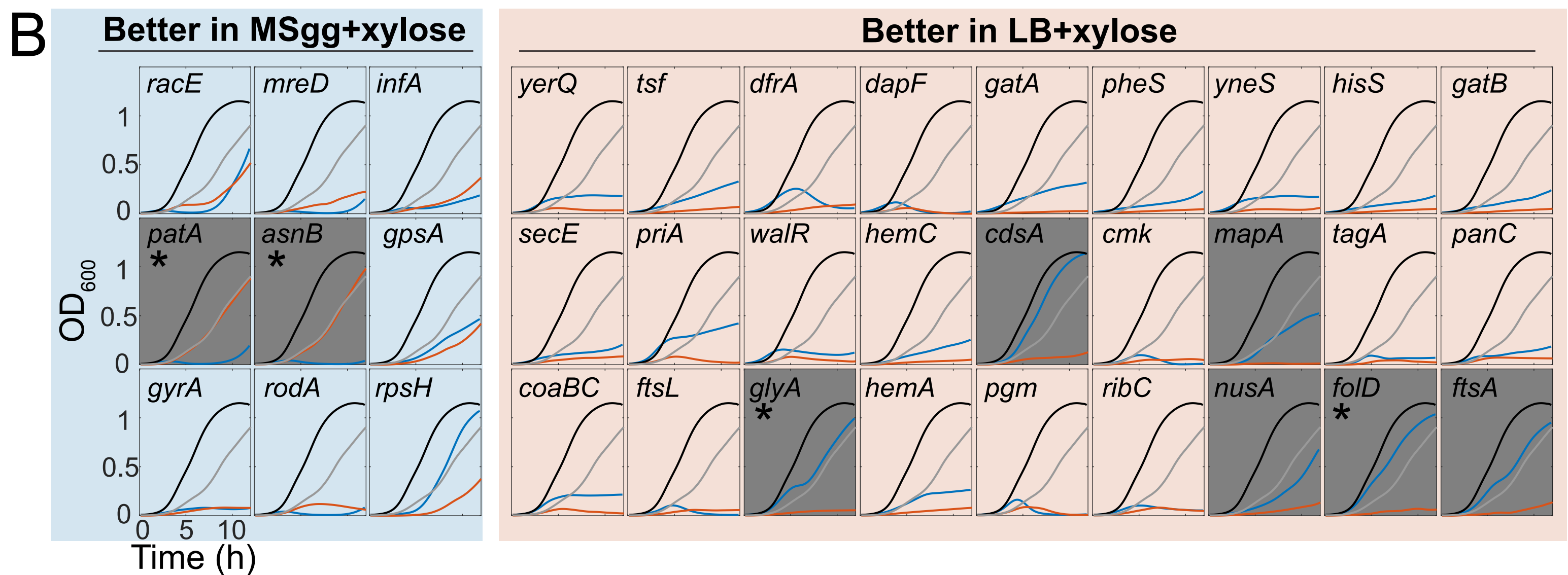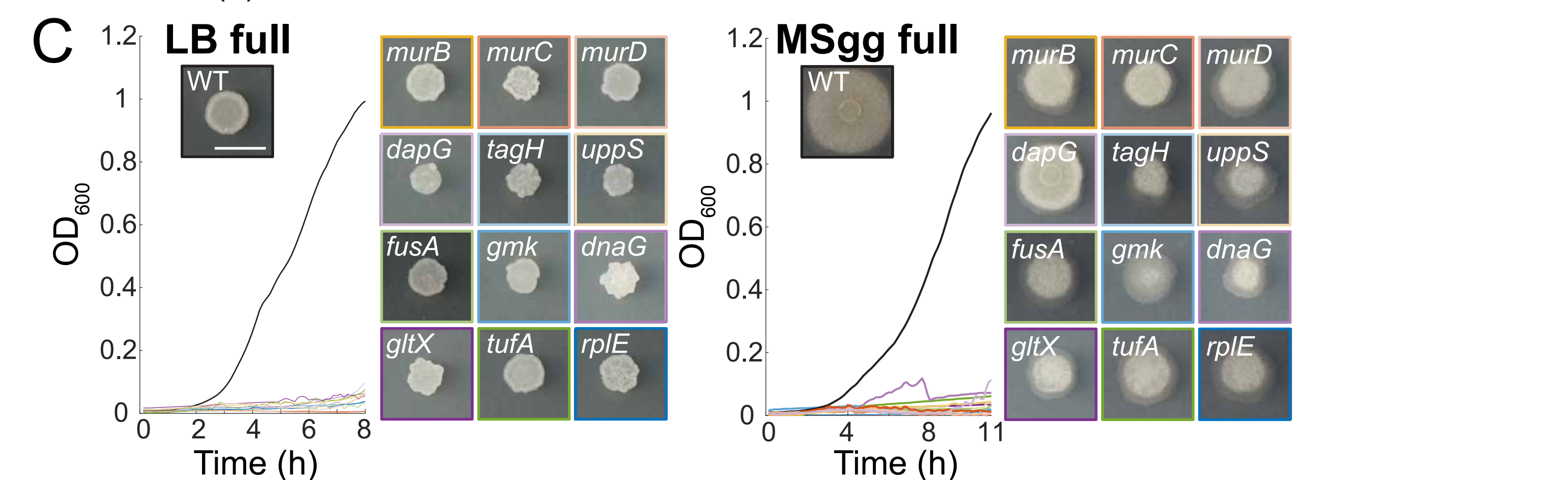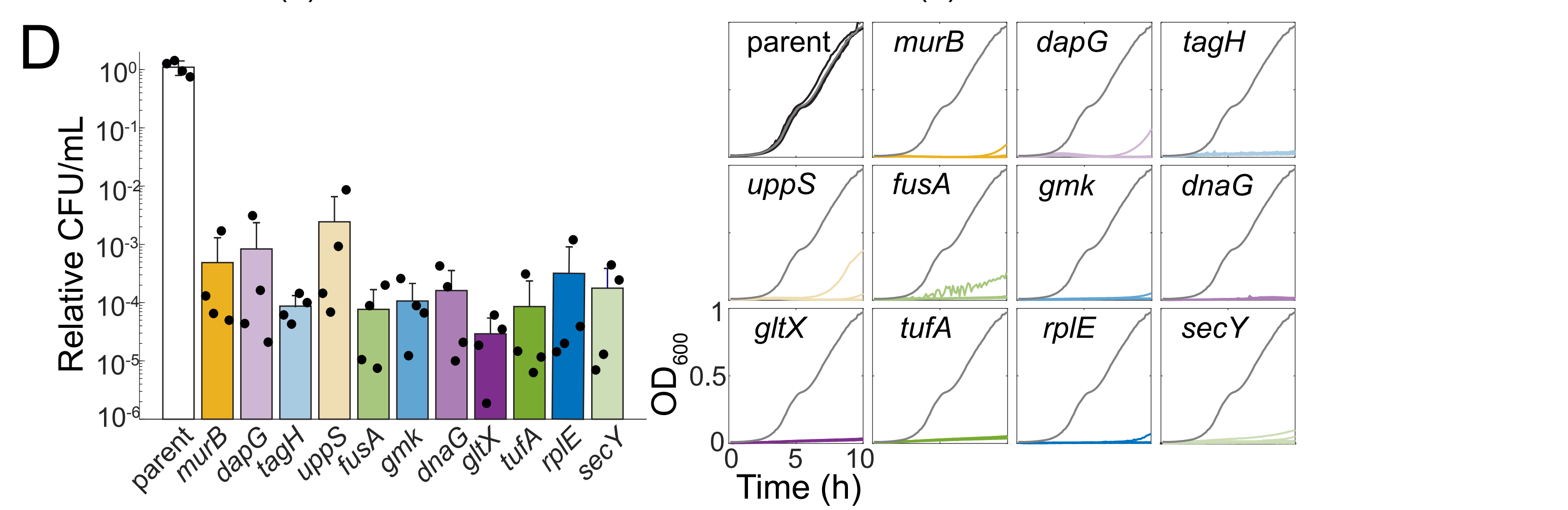

Supplement: FIG S2 [file mbio.01388-22-s0008.pdf]

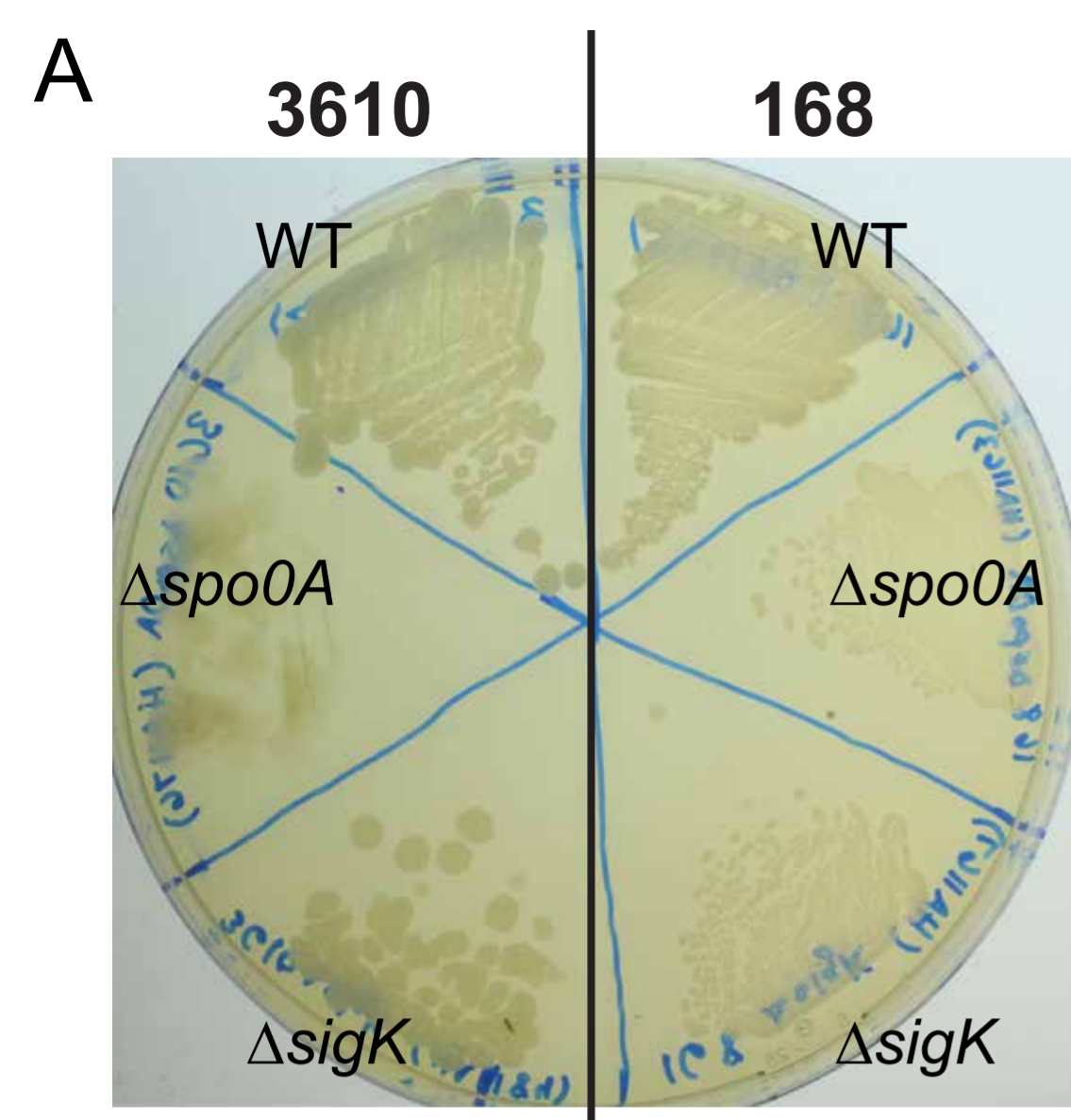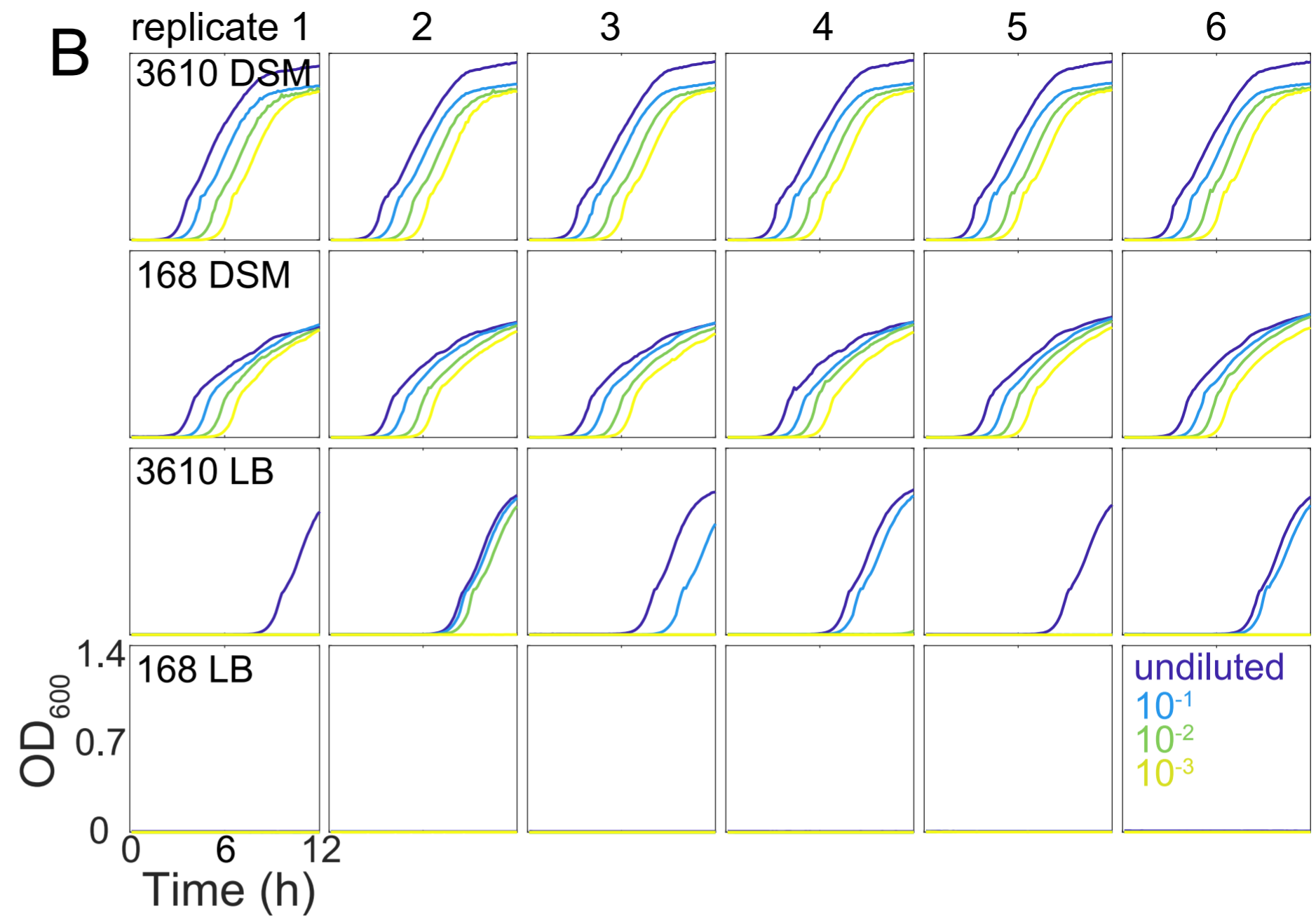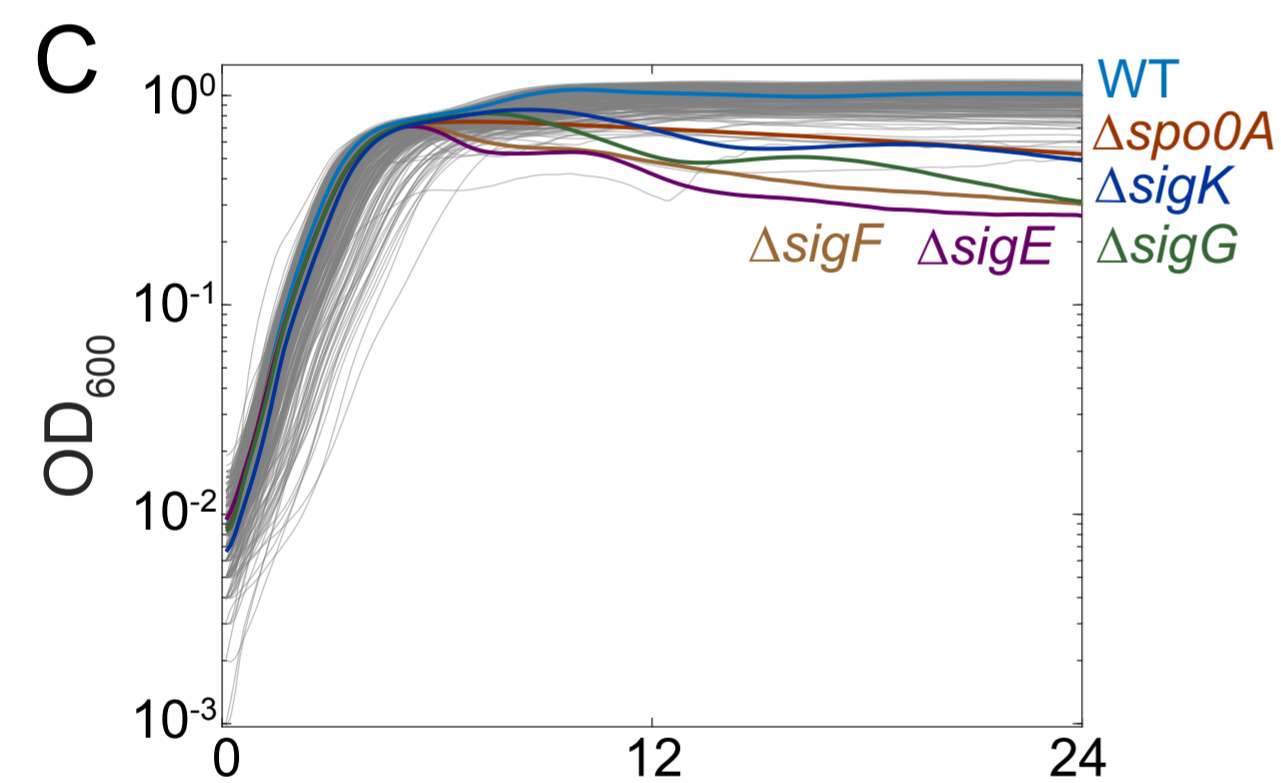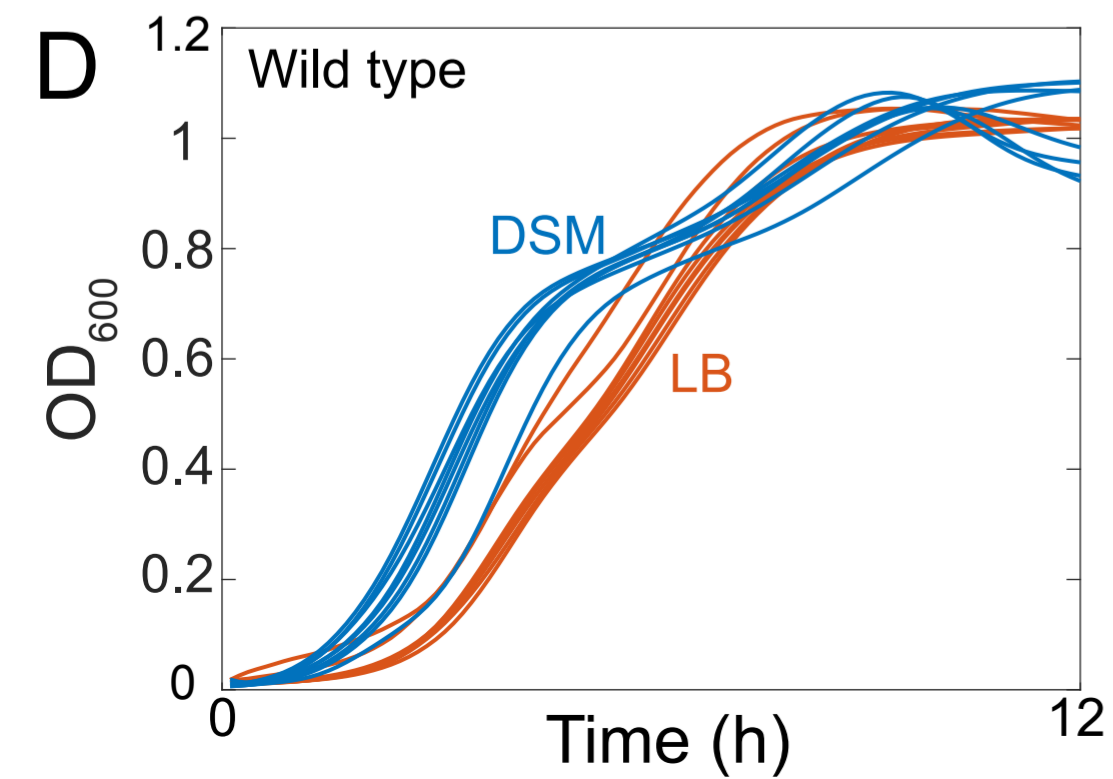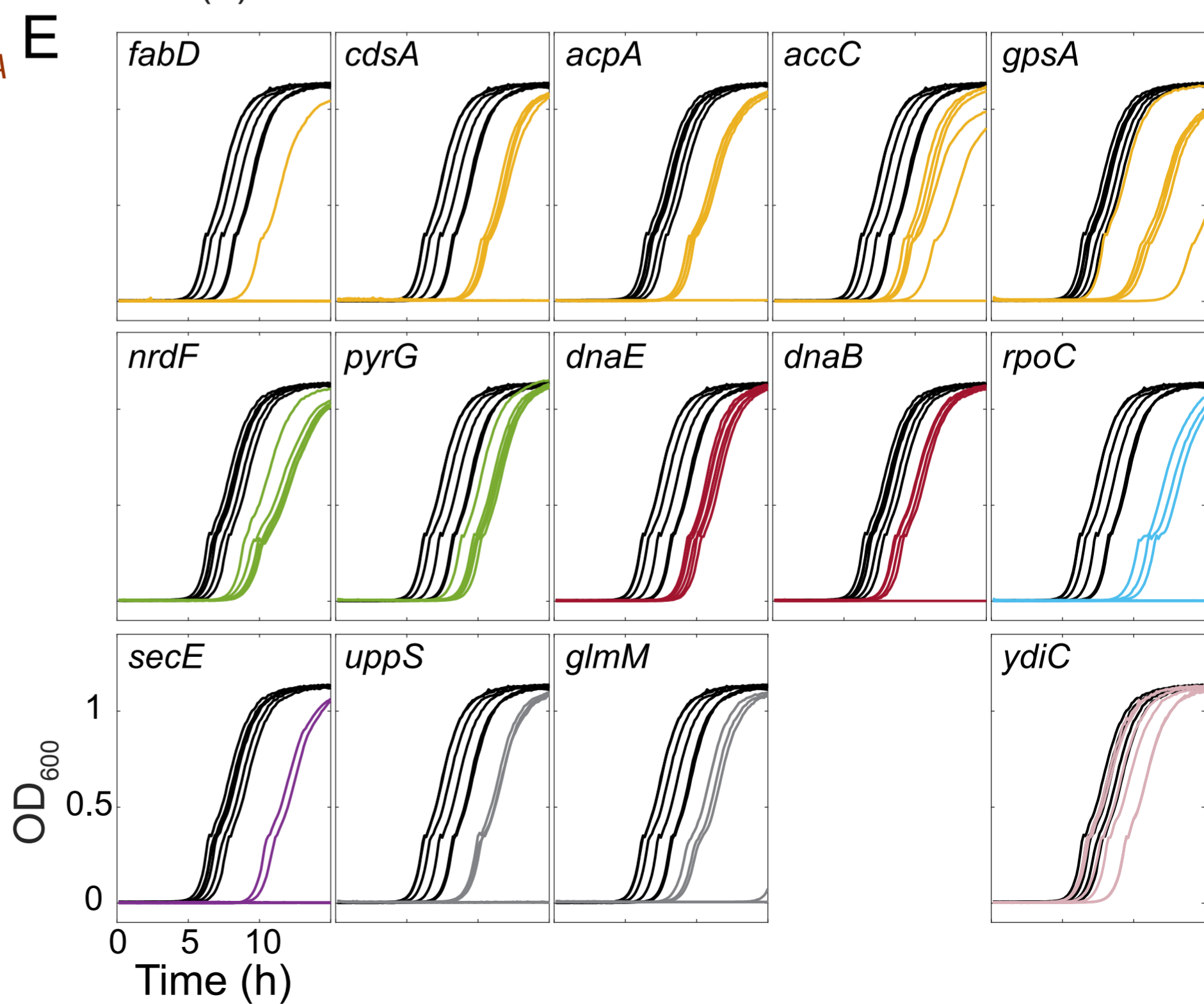

Supplement: FIG S4 [file mbio.01388-22-s0010.pdf]
