# Supplementary material for: Fatty Acid Synthesis Knockdown Promotes Biofilm Wrinkling and Inhibits Sporulation in Bacillus subtilis
Source: mBio. 2022 Sep 7;13(5):e01388-22. doi: 10.1128/mbio.01388-22 (PMC9600695; doi:10.1128/mbio.01388-22)
Supplement: FIG S3 [file mbio.01388-22-s0009.pdf]

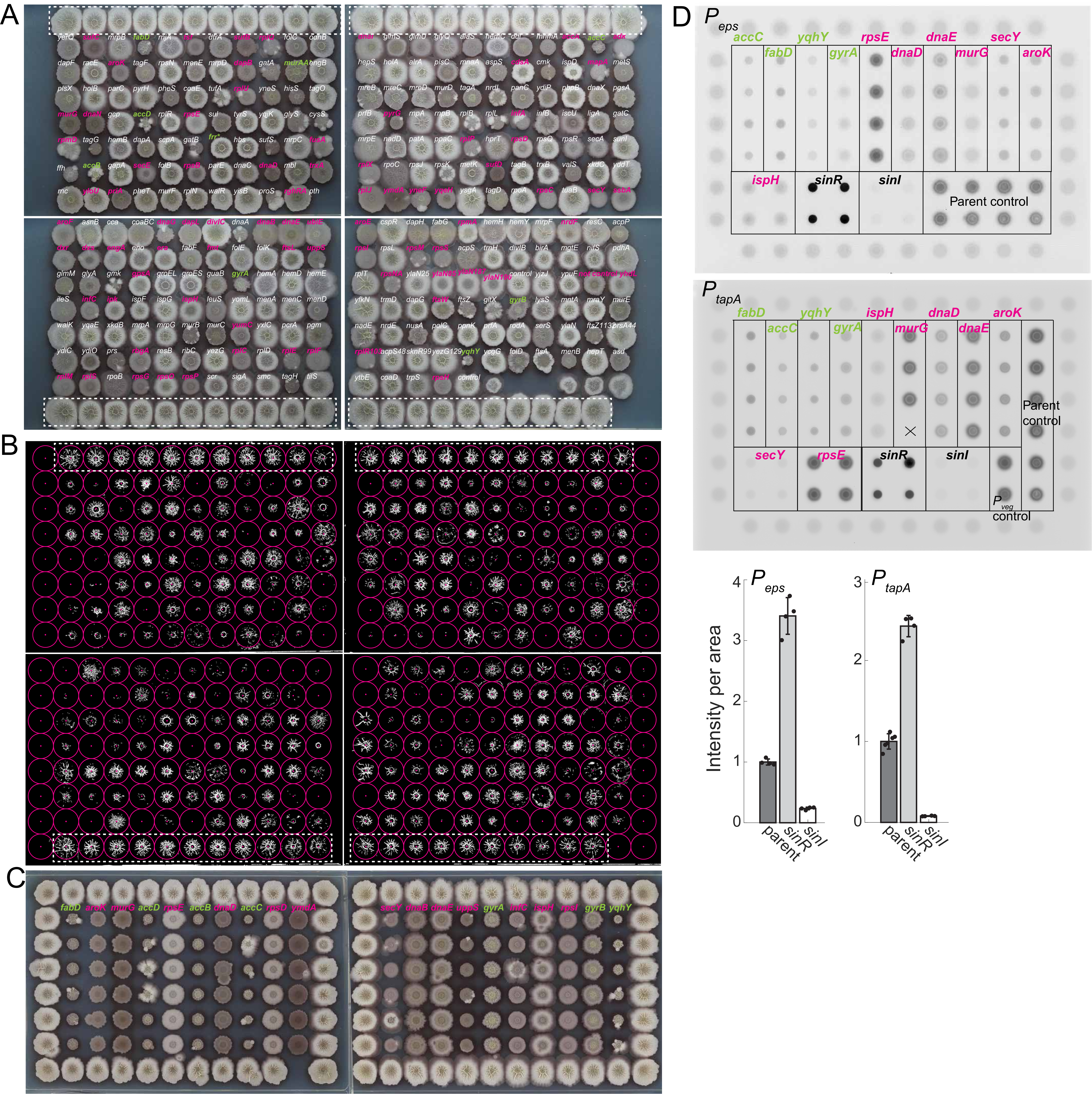
A
yerQ
mrpB
fabD
rnjA
dfrA
folC
odhB
glmS
glmU
glyQ
alaS
hemC
def
mnmA
accC
adk
dapF
racE
aroK
tagF
rpsN
menE
mrpD
gatA
murAA
engB
hepS
holA
alrA
plsC
mnaA
aspS
cmk
ispD
metS
plsX
holB
parC
pyrH
pheS
coaE
tufA
yneS
hisS
tagO
mreB
mreC
mreD
murD
tagA
nrdI
panC
ydiP
pbpB
dnaX
pgsA
murG
dnaN
gcp
accD
rplR
rpsE
sul
tyrS
yqjK
glyS
cysS
prfB
pyrG
rnpA
rnpB
rplB
rplL
infB
iscU
ligA
gatC
tagG
hemB
dapA
scpA
gatB
frr*
hbs
sufS
mrpC
fusA
mrpE
nadD
patA
ppaC
hprT
rpsQ
rpsR
secA
sunI
ffh
accB
gapA
folB
parE
dnaC
dnaD
mbl
rpoC
rpsJ
rpsK
metK
tagB
trxB
valS
xkdC
yddT
rnc
pheT
murF
rplN
walR
yisB
proS
pth
ysgA
tagD
rpoA
rpsC
tuaB
secY
ssbA
asnB
cca
coaBC
dnaG
divIC
dnaA
dnaB
dnaE
cspR
dapH
fabG
hemH
hemY
mrpF
resC
acpP
eno
fabF
folE
folK
uppS
rpsL
acpS
trnH
divIB
birA
mgtE
nifS
pdhA
glmM
glyA
gmk
groEL
groES
guaB
gyrA
hemA
hemD
hemE
rplT
ylaN25
ylaN85
ylaN197
ylaN190
control
yjzJ
ypuF
not control
ileS
infC
ipk
ispF
ispG
ispH
leuS
yomL
menA
menC
menD
yfkN
trmD
dapG
ftsZ
gltX
gyrB
lysS
mntA
mraY
murE
walK
yqaE
xkdB
mrpA
mrpG
murB
murC
yumC
yxlC
pcrA
pgm
nadE
nrdE
nusA
polC
ppnK
prfA
rodA
serS
ylaN
ftsZ1132
prsA44
ydiC
ydiO
prs
resB
ribC
yezG
rplD
acpS48
sknR99
yezG129
yqhY
ycgG
folD
ftsA
menB
hepT
asd
rplM
rplS
rpoB
rpsG
rpsO
rpsP
scr
sigA
smc
tagH
tilS
ytbE
coaD
trpS
control
B
C
fabD
aroK
murG
accD
rpsE
accB
dnaD
accC
rpsD
ymdA
secY
dnaB
dnaE
uppS
gyrA
infC
ispH
rpsI
gyrB
yqhY
D
P_{eps}
accC
fabD
yqhY
gyrA
rpsE
dnaD
dnaE
murG
secY
aroK
ispH
sinR
sinI
Parent control
P_{tapA}
fabD
accC
yqhY
gyrA
ispH
murG
dnaD
dnaE
aroK
Parent control
secY
rpsE
sinR
sinI
P_{veg} control
P_{eps}
P_{tapA}
Intensity per area
0
1
2
3
4
parent
sinR
sinI
